# The case for etanercept in the management of toxic epidermal necrolysis

**DOI:** 10.1093/skinhd/vzaf012

**Published:** 2025-05-28

**Authors:** Encarl Uppal, Kate Dear, Lesley Exton, David Ryan, Christopher B Bunker

**Affiliations:** Department of Dermatology, University College London Hospitals NHS Foundation Trust, London, UK; Department of Dermatology, University College London Hospitals NHS Foundation Trust, London, UK; Clinical Standards Unit, British Association of Dermatologists, London, UK; Department of Clinical Pharmacology and Therapeutics, University College London Hospitals NHS Foundation Trust, London, UK; Department of Dermatology, University College London Hospitals NHS Foundation Trust, London, UK

## Abstract

Toxic epidermal necrolysis (TEN) is a dermatological emergency with devastating morbidity and mortality rates. It is most often caused by medications. Management guidelines have focused on conservative measures, partly due to a previous lack of large randomized controlled trials evaluating systemic agents, and because experts have differing views on initiating active medical management. Tumour necrosis factor alpha (TNF-α) is a cytokine that is involved in immune defence against various infectious agents by exerting a powerful anti-inflammatory effect. Inhibition of TNF-α is well established in the management of noninfectious inflammatory (including dermatological) conditions. Patients with TEN have prominent expression of TNF-α in keratinocytes and macrophages in affected skin as well as in blister fluid. This review article aims to summarize the recent and emerging evidence for using anti-TNF-α treatment, particularly etanercept, in the management of TEN.

## Background

Toxic epidermal necrolysis (TEN), although rare, is the most severe of the cutaneous drug reactions with a mortality rate of approximately 30%, rising to 50% in older patients and those who are immunocompromised.^1^ It is characterized by widespread keratinocyte apoptosis and detachment of the epidermis and is primarily caused by drugs including antibiotics, anticonvulsants and nonsteroidal anti-inflammatory drugs. The acute phase may be accompanied by a variety of systemic complications including multiorgan failure. If patients survive, they are at a high risk of permanent disability and long-term sequalae.

There have been few large, randomized controlled trials that have involved systemic treatment for TEN; therefore, much of the literature reporting benefits of various drugs is limited to case reports and series. Indeed, to date, no systemic treatments are recommended in British guidelines;^2^ however, there is known to be considerable variation in practice, with many experienced clinicians favouring active medical management, often with several drugs. It seems likely that as more positive evidence becomes available about medical interventions, including etanercept, that this will be reflected in fresh iterations of guidelines. However, guidelines take a long time to emerge, one reason for our preparation of this interval contribution.

Current treatment for Stevens–Johnson Syndrome (SJS)/TEN (as delineated by the 2016 UK British Association of Dermatologists guidelines by Creamer *et al.* 2016),^2^ focuses on supportive management, with surgical management (debridement and physical closure with Biobrane/allograft/xenograft skin) in certain cases. Regarding active medical management, the guidelines state ‘currently, no active therapeutic regimen with unequivocal benefit exists for Stevens–Johnson Syndrome (SJS)/TEN. … [however] the immunological basis for SJS/TEN has led physicians to prescribe immunomodulating drugs, several of which have been reported in uncontrolled series’. Of these drugs, the only ones that met the inclusion criteria for consideration in 2016 were intravenous immunoglobulin (IVIG), systemic corticosteroid and ciclosporin. However, the possibility of an emerging role for etanercept was favourably discussed (based on a small case series published by Paradisi *et al.*^3^). Overall, the authors ‘did not consider any of the [then available data] of sufficient quality or consistency to make specific recommendations either for or against the use of active interventions, and [they] highlighted the need for future research’. It was noted that there was a ‘lack of consensus even among clinicians with experience in managing SJS/TEN [and the authors concluded]… [that] if active therapy is instituted it should be given, ideally, under the supervision of a specialist skin failure multi-disciplinary team in the context of clinical research and/or case registry’. Since then, many clinicians and teams have exhibited active treatment with individual agents or combinations of agents. Etanercept has grown in use as clinicians have become experienced in its efficacy and safety, and as the literature supporting its efficacy and safety has grown.

Etanercept is a tumour necrosis factor alpha (TNF-α) inhibitor. TNF-α is a cytokine that exerts powerful inflammatory effects on the immune system’s defence against various infectious agents. At the cellular level, TNF-α can lead to cell death through direct cytotoxicity and induction of apoptosis, and exposure of healthy explanted skin to TNF-α can induce epidermal detachment.^4^ Blocking or neutralizing TNF-α has proven effective in treating noninfectious inflammatory conditions, including Crohn disease, rheumatoid arthritis and psoriasis. In patients with SJS/TEN, TNF-α is prominently expressed in keratinocytes and macrophages in the affected skin, with high concentrations found in serum, blister fluid and skin of patients with SJS/TEN compared with controls.^5^ Etanercept has been shown to effect a decrease in TNF-α secretion in blister fluid and plasma.^6^ However, many cytokines and cytotoxic proteins are present at higher concentrations in blister fluid/skin tissue; and it is unclear which proteins are pathogenic, diagnostic and/or markers of disease severity and course.^5^

## Methods

A systematic literature search of the ProQuest Dialog, MEDLINE, Embase and Cochrane databases was conducted by the British Association of Dermatologists technical team to identify key articles according to their protocol for the update of their 2016 SJS/TEN guideline^2^ from 1 January 1990 up to 25 January 2024. Articles published before 1990 were excluded because the internationally accepted consensus definition for diagnosing SJS/TEN was developed in 1990.^7^ The identified case reports part of their ProQuest Dialog, MEDLINE and Embase database search which were outside their guideline protocol have been included in this work, along with appropriate key articles, for example systemic reviews, case series. Search terms and strategies are detailed in Table 1.

**Table 1:** Search strategy; duplicates were removed from search and result count in MEDLINE/Embase database (search carried out on 19 January 2023; top-up 25 January 2024)

Search no.	Keywords
1	meta-analysis OR meta-analyses OR meta pre/0 analysis OR meta pre/0 analyses OR EMB.EXACT.EXPLODE(‘meta analysis’) OR (systematic pre/0 review$1) OR EMB.EXACT(‘systematic review’) OR (randomized pre/0 control$3 pre/0 trial$1) OR (randomized pre/0 control$3 pre/0 trial$1) OR RCT$1 OR (randomized pre/0 control pre/0 trial) OR (randomized pre/0 control pre/0 trial) OR EMB.EXACT.EXPLODE(‘randomized controlled trial’) OR (control$3 pre/0 trial$1) OR (control$3 pre/0 clinical pre/0 trial$1) OR (clinical pre/0 monitor$3) OR EMB.EXACT.EXPLODE(‘controlled clinical trial’) OR (case pre/0 series) OR (case pre/0 control$1) OR (open pre/0 stud$3) OR (cohort pre/0 stud$3) OR MESH.EXACT.EXPLODE(‘Case-Control Studies’) OR EMB.EXACT.EXPLODE(‘case control study’) OR EMB.EXACT.EXPLODE(‘clinical study’) OR retrospective PRE/0 stud$3 OR MESH.EXACT.EXPLODE(‘Retrospective Studies’) OR EMB.EXACT.EXPLODE(‘retrospective study’)
2	(case pre/0 report$1) OR EMB.EXACT(‘case report’)
3	1 NOT 2
4	EMB.EXACT(‘toxic epidermal necrolysis’) OR (toxic pre/0 epidermal pre/0 necrolysis) OR (toxic pre/0 epidermal pre/0 necrosis) OR ti,ab(toxic epidermal necro$5) OR (epidermal pre/0 necrosis) OR (epithelial pre/0 necrosis) OR (epidermal pre/0 necrolysis)
	**Cochrane**: (toxic epidermal necrolysis):ti,ab,kw OR (epidermal necrosis):ti,ab,kw OR (epithelial necrosis):ti,ab,kw OR (epidermal necrolysis):ti,ab,kw OR (toxic epithelial necrosis):ti,ab,kw
5	EMB.EXACT(‘Stevens Johnson syndrome’) OR MESH.EXACT(‘Stevens-Johnson Syndrome’) OR (stevens-johnson pre/0 syndrome) OR (stevens pre/0 johnson pre/0 syndrome) OR ti,ab(Stevens Johnson Syndrome) OR ti,ab(Stevens-Johnson Syndrome)
	**Cochrane**: [mh ‘Stevens-Johnson Syndrome’] OR (Stevens-Johnson Syndrome):ti,ab,kw
6	ti(Lyell$2) OR ab(Lyell$2)
7	4 OR 5 OR 6
	Cochrane: 4 OR 5
8	3 AND 7
9	Limit date to publications from 01.01.1990
	**Cochrane**: Limit date to publications from 01.01.1990

## Discussion

The literature has seen a growing body of case reports that report benefit from anti-TNF-α treatment in TEN following the use of infliximab,^1,8–11^ adalimumab^12^ and more recently, etanercept,^13–34^ with cessation of epidermal detachment and subsequent re-epithelialization of the skin. Furthermore, at a cellular level, anti-TNF-α therapy has shown to markedly decrease TNF-α in lesional skin. Etanercept has several advantages over infliximab, including subcutaneous instead of intravenous administration, thus eliminating infusion reactions and avoiding the risk of volume overload. While patients receiving these potent immunosuppressants must be monitored for signs of invasive infection, etanercept seems to carry a lower risk of activating latent tuberculosis and invasive fungal infections than infliximab.^35,36^ There is even less evidence about adalimumab use in TEN, making comparison challenging. We hypothesize that the preferred use of etanercept in the treatment of SJS/TEN over more recent anti-TNF-α medications is down to lack of availability of such biologics in earlier trials, when the medication was first being trialled. Furthermore, infliximab and adalimumab have a longer half-life than etanercept,^37^ increasing duration of exposure and potential infection risk in patients with TEN, a risk perhaps physicians were earlier unwilling to accept. Etanercept is also less expensive than both infliximab and adalimumab,^38^ improving accessibility. The subsequent success of treatment and proven safety of etanercept then likely led to further prescribing over more recent anti-TNF-α drugs. However, it is possible that another anti-TNF agent could be equally or more effective with a similar adverse effect profile. This could be due to the differing mechanisms of action between a fusion protein (etanercept) and monoclonal antibody (infliximab and adalimumab).

Early studies reporting the benefit of etanercept in TEN include a case series by Paradisi *et al.* in 2014,^3^ who described a rapid and complete response with complete re-epithelialization in 10 patients with TEN treated with one dose of etanercept, without complications or side-effects. In a different study the same authors reported 17 patients who had received a single injection of etanercept 50 mg.^39^ Patients were assessed according to the widely used Severity-of-Illness Score for Toxic Epidermal Necrolysis (SCORTEN) criteria, and the lowest score was 2, with seven patients having SCORTEN >5. Two patients in the highest risk category (SCORTEN >4) died. A total of 15 patients responded rapidly to treatment and achieved complete re-epithelialization (median time to healing 8.5 days) with no complications or adverse events reported.

Zimmerman *et al.* conducted a systematic review of systemic therapies for SJS/TEN in 2015 that included 3248 patients.^40^ Corticosteroids and ciclosporin were the only two therapies associated with increased survival. However, studies reporting on five or fewer patients were excluded and the authors acknowledged that TNF-α inhibitors (specifically etanercept) had appeared in some such studies and case reports of SJS/TEN that were excluded from their analysis.

In a 2018 review of published case reports and case series between 1994 and 2014 (*n* = 28), Woolridge *et al.* found in favour of the use of TNF-α inhibitors in TEN in adult and paediatric populations (albeit cautioning about the risk of infection).^35^ Also in 2018, Wang *et al.* reported a randomized trial of 96 patients with SJS/TEN, comparing etanercept (25 mg twice weekly or 50 mg if weighing > 65 kg) with corticosteroids (prednisolone 1–1.5 mg kg^−1^ daily).^6^ This study showed that etanercept improved clinical outcomes, with a decrease in the SCORTEN-based predicted mortality rate (predicted and observed rates 17.7% and 8.3%, respectively), a reduction in skin-healing time in patients with moderate-to-severe SJS/TEN patients (median time for skin healing was 14 and 19 days for etanercept and corticosteroids, respectively; *P* = 0.01) and a lower incidence of gastrointestinal haemorrhage in all patients with SJS/TEN (2.6% for etanercept and 18.2% for corticosteroids; *P* = 0.03). The incidence of adverse events was also evaluated, with 6 serious adverse events in 5 patients in the etanercept group vs. 12 in 9 patients in the corticosteroid group. These complications were considered not to be related to etanercept or cortico­steroid treatment.

A systematic review conducted by Sachdeva *et al.* found 38 articles regarding patients diagnosed with SJS/TEN and treated with biologics.^41^ Apart from finding that anti-TNF-α therapy, specifically etanercept, showed improved outcomes as monotherapy, they also remarked that biologic monotherapy may be safer than combination therapy with corticosteroids, IVIG or ciclosporin.

Tsai *et al.* performed a systematic review and network meta-analysis of 67 studies involving 2079 patients and ranked etanercept the best (of 10) among systemic treatments when assessing mortality rate by ‘surface under the cumulative ranking curve’ (SUCRA).^42^ Furthermore, etanercept and the combination of corticosteroids and IVIG were the first two ranked therapies (among eight treatments) according to their SUCRA. However, the studies included had small sample sizes, and estimates of effect were not statistically significant for standardized mortality rate (SMR), owing to wide confidence intervals. Thus, the study concluded that a combination of corticosteroids and IVIG was the only treatment with significant survival benefits by SMR; however, the authors concluded that etanercept was a promising therapy in need of further research to provide clearer evidence.

Dreyer *et al.* retrospectively compared 13 patients treated with etanercept and 9 patients treated with IVIG or supportive care alone between 2013 and 2016;^43^ 9 patients received etanercept alone and 4 patients received etanercept and IVIG. The observed mortality rate was 0% in the etanercept group vs. 33% in the IVIG or supportive care group. Two patients progressed despite the administration of etanercept and were then successfully treated with IVIG. Patients treated with etanercept also had a faster time to re-epithelialization (3–19 vs. approximately 14–21 days).

Only 7 patients treated with etanercept were included in the pan-European ToxiTEN group longitudinal, retrospective, cohort study of 212 patients, so no useful data can be adduced.^44^

A 2021 Canadian retrospective study by Olteanu *et al.* included 43 patients with SJS/TEN treated over a 16-year period; 3 were treated with etanercept in combination with other systemic agents, none of whom died (total number of deaths in the study was 9).^45^

A Taiwanese group retrospectively reviewed patients treated at their centre with severe ocular complications of SJS/TEN, as assessed by the Sotozono 2007 grading system. Between 2010 and 2020, 31 patients received combination corticosteroid and etanercept and 82 patients received corticosteroid only. Those treated with etanercept displayed better final vision and significantly lower mean (SD) chronic SJS/TEN grading scores: 1.64 (2.47) (range 0–14) vs. 3.95 (5.76) (range 0–33) (*P* < 0.001). There was no significant difference in the acute-stage grading.^46^

In 2022, a study by Ao *et al.* compared 10 patients receiving methylprednisolone (1–1.5 mg kg^−1^ daily) with 15 patients receiving etanercept (25 mg twice weekly until steroid tapering) plus methylprednisolone (1–1.5 mg kg^−1^ daily). The combination group required a shorter duration of systemic steroids and a shorter duration of the acute stage of SJS/TEN, and had a shorter period of hospitalization and faster skin re-epithelialization.^4^ A pooled meta-analysis by Krajewski *et al.* in the same year reviewed 42 studies assessing systemic treatment for SJS/TEN and found that lowest mortality rate to be linked to etanercept.^47^ A Cochrane review, also in 2022, which included 9 studies (including 308 individuals), found that when compared with corticosteroids, etanercept may reduce the number of deaths in patients with SJS/TEN.^48^ A retrospective cohort study of 41 patients by Tian *et al.* found that etanercept reduced the overall dose and duration of systemic steroids needed and the duration of hospitalization.^49^ Importantly, no patients experienced any adverse effects of etanercept therapy during admission or within 6 months of follow-up. Furthermore, a large study by Zhang *et al.* (including 242 patients) showed that combination therapy with etanercept and corticosteroids resulted in a lower mortality rate than with corticosteroids alone or combination therapy of IVIG and corticosteroids.^50^ There were few side-effects of etanercept treatment and the authors found the incidence of gastrointestinal haemorrhage to be lower in the etanercept + corticosteroid group than in the corticosteroid monotherapy or IVIG and corticosteroid group.

A more recent meta-analysis by Cao *et al.* found that etanercept had a lower mortality rate than the average reported rate in epidemiological studies in patients with SJS/TEN. It further showed that etanercept therapy provided comparable survival outcomes and shorter hospital stays than nonetanercept treatments.^51^ It should be noted that the two case series by Paradisi *et al.* totalling 27 patients treated with etanercept found no severe adverse effects,^3,39^ and no severe side-effects were described in the 23 case reports comprising 26 patients, including children and immunosuppressed patients and those with other comorbidities. However, three fatalities have been reported by one group, but the mortality rate for TEN is high and the cause could not be proven.^52^

Table 2 provides a summary of the key papers and their findings.

**Table 2:** Summary of results from key papers ordered by date of publication and alphabetization of primary author surnames

Study	Series	Dates studied	Key study details	Key results	Adverse events
Paradisi, 2014^3^	Uncontrolled case series	2011–2012	*n* = 10, etanerceptExpected probability of death by SCORTEN scores; 46.9%	10/10 patients reached re-epithelization without complications or side-effects (95% CI for the observed MR (i.e. 0) ranging from 0% to 30.8%)Complete re-epithelialization time; 7–20 days (median: 8.5 days)	Nil specific reported
**Wang, 2018** ^6^,^a^	RCT	2009–2015	*n* = 48, etanercept*n* = 45, corticosteroidsMean SCORTEN for etanercept and corticosteroid groups = 1.85 vs. 1.95 (*P* = 0.72)SCORTEN-predicted MRs for the etanercept and corticosteroid groups = 17.7% vs. 20.3%Authors also completed retrospective review of a retrospective cohort of CTL-mediated patients with SCAR treated with supportive care 2001–2008; *n* = 38, MR = 26.3%	71 participants completed study: *n* = 38 etanercept, *n* = 33 corticosteroidsMR for the etanercept vs. corticosteroid group; 8.3% vs 16.3% (*P* = 0.27)Etanercept reduced SCORTEN-based predicted MR (predicted and observed rates, 17.7% and 8.3%, respectively)MR of the etanercept treatment group was significantly lower than that of the supportive care group (*P* = 0.03, by unconditional z-pooled test)No statistical difference between in MR between corticosteroid group and supportive care group (*P* = 0.28)Median time for skin healing; etanercept vs. corticosteroid = 14 vs. 19 days (*P* = 0.01)Etanercept decreased TNF-α and granulysin secretions in blister fluids and plasma (45.7–62.5% decrease after treatment; all *P* < 0.05) and increased Treg population (2-fold percentage increase after treatment; *P* = 0.002), which was related to mortality in severe SJS/TEN	Incidence of GI haemorrhage; etanercept vs. corticosteroids = 2.6% vs. 18.2% (*P* = 0.03)Six SAEs in 5 participants in the etanercept group vs. 12 SAEs in 9 in the corticosteroid group, e.g. sepsis, respiratory failure, upper (GI) haemorrhage (grade 3), bipolar disorder, stridor and vocal cord palsyEleven patients died, 4 of whom had received etanercept, 7 of whom had received corticosteroid treatment
Paradisi, 2020^39^	Case series	2014–2016	*n* = 17, etanerceptSCORTEN scores ranged from 3–7, 7 patients scored in the most severe category (> 5)	Observed MR (*n* = 2/17,11.8%) vs. expected probability of death by SCORTEN scores (*n* = 10/17, 58.8%), was statistically significant (*P* = 0.01)Fifteen achieved complete re-epithelization (median time to healing: 8.5 days), without complications or side-effects	Two observed deaths, both due to other causes Re-epithelization had initiated in both patients
**Dreyer, 2021** ^43^,^a^	Retrospective case series	2013–2016	*n* = 9, etanercept monotherapy*n* = 4, etanercept + IVIG*n* = 5, IVIG monotherapy*n* = 4, supportive care	Observed MR = 0%Etanercept group; mean SCORTEN = 2, predicted mortality = 22.9%Etanercept + IVIG group; mean SCORTEN = 2.3, predicted mortality = 27.2%IVIG was given for progression of disease despite administration of etanercept in 2 patients. The 2 other patients on combination treatment were given combination treatment due to the perceived severity at presentationIVIG monotherapy and supportive care groups (*n* = 9); mean SCORTEN = 2.4, predicted MR = 28.1%, observed MR = 33%Re-epithelialization data available for 8 etanercept patients (*n* = 6 etanercept monotherapy, *n* = 2 etanercept + IVIG); median = 8.9 days (3–19 days)Re-epithelialization data not available for IVIG monotherapy or supportive care groups	Nil specific reported
Ma, 2021^46^	Retrospective	2010–2020	SJS *n* = 87, SJS/TEN *n* = 9, TEN *n* = 23*n* = 31, corticosteroid + etanercept*n* = 82, corticosteroid monotherapyNo significant difference in sex, age, distribution of culprit drugs, severity of acute ocular complications and follow-up time between patients receiving corticosteroid monotherapy and patients receiving corticosteroid + etanercept	Those treated with etanercept displayed better final vision and significantly lower chronic SJS/TEN grading scores: 1.64 ± 2.47 (0–14) vs. 3.95 ± 5.76 (0–33),_b_ *P* < 0.001	Nil specific reported
**Sachdeva, 2021** ^41^,^a^	Systematic review	October 2020	38 articles reviewed, 27 (71.1%) case reports, 6 (15.8%) case series, 3 (7.9%) retrospective reviews and 2 (5.3%) RCTsSources were classified as either biologic monotherapy or combination therapy; defined as a biologic therapy used in combination with other systemic therapies (ex–*N*-acetylcysteine, systemic corticosteroids, immunoglobulin)*n* = 67, etanercept monotherapy*n* = 9, infliximab monotherapy*n* = 27, etanercept combination group*n* = 16, infliximab combination group	Mean BSA across all articles was 31.0%, average actual mortality 9.2%Average number of days to re-epithelization in treatment with etanercept monotherapy was 13.1 days and the average actual MR was 7.6%Average number of days to re-epithelization was 10.4 days in infliximab monotherapy, no cases of mortalityAverage number of days to re-epithelization in combined therapy with etanercept was 11.1 days and the average actual MR was 7.7%Average number of days to re-epithelization in combined therapy with infliximab was 14.2 and the average actual MR was 22.2%Biologic monotherapy and combination therapy were associated with improved outcomes in SJS/TENAnti-TNF-α therapy, specifically etanercept, showed improved outcomes as monotherapy	Nil specific reported
Tsai, 2021^42^	Systematic review and NMA	October 2019	67 studies, *n* = 2079 patients	In the NMA of 10 treatments, no treatment was significantly superior to supportive care concerning the MRAccording to the SUCRA, for mortality, etanercept was ranked the best among the 10 treatments, followed by a combination of corticosteroids and IVIG	Nil specific reported
Ao, 2022^4^	Nonrandomized active treatment control	2017–2021	*n* = 10, methylprednisolone*n* = 15, etanercept + methylprednisoloneNo significant difference in SCORTEN scores in both groups	All 25 patients were cured without any sequelaEtanercept + methylprednisolone significantly shortened skin healing time (median, 12 days) compared with steroid monotherapy group (median, 16 days (*P* = 0.01)Etanercept + methylprednisolone therapy significantly shortened the course of the initial steroid treatment (*P* = 0.004), the duration of the acute stage (*P* = 0.001) and hospitalization stay (*P* = 0.02)	Post-treatment infections occurred in 5 patients who received steroid monotherapy and in 3 patients treated with the etanercept +methylprednisolone therapy
Cochrane review, 2022^48^	Systematic review	March 2021	9 (3 RCTs and 6 prospective, controlled observational studies), total of 308 participants (131 male and 155 female patients) from 7 countries included^c^	No studies met the criteria for evaluation of etanercept to supportive care, IVIG or ciclosporinEtanercept may reduce disease-specific mortality compared to corticosteroids (RR 0.51, 95% CI 0.16–1.63; 1 study; 91 participants; low-certainty evidence)	SAEs (e.g. sepsis and respiratory failure), reported in 5 of 48 participants with etanercept and 9 of 43 participants with corticosteroids, but it was not clear if they led to discontinuation of therapy
Krajewski, 2022^47^	Metanalysis and meta-regression of observational studies	March 2020	41 observational studies and 1 RCT with a total of *n* = 1942 patients	Using random-effects model, the overall pooled MR was 0.191 (95% CI 0.132–0.269). The lowest MR was found to be linked with etanercept use (*n* = 2); pooled MR of 0.073 (95% CI 0.018–0.254), followed by ciclosporin treatment (*n* = 8); pooled MR of 0.086 (95% CI 0.0421–0.169)Highest MR seen with TPE and IVIGOverall re-epithelization was 13.278 days (95% CI 8.773–17.784), the highest was found in ciclosporin treatment (14.739), while the lowest for steroidsDuration of hospital stay in overall analysis was 19.99 days (95% CI 16.53–23.44), the highest was linked with TPE/TPE + IVIG treatment, the lowest with steroids	Nil specific discussed
Tian, 2022^49^	Retrospective	2015–2021	*n* = 14, IVIG and corticosteroids*n* = 11, IVIG, corticosteroids and etanerceptSCORTEN-predicted MRs were 12.2% in both groups	Conjunction with etanercept reduced the duration of hospitalization (13.5 vs. 19.0 days; *P* = 0.01), the exposure time of high-dose steroids (7.1 vs. 14.9 days; *P* = 0.01) and the overall amount of systemic steroid (925 mg vs. 1412.5 mg; *P* = 0.03)No significant difference noted between the 2 groups regarding the cumulative dose of IVIG (110.5 vs. 125.0 g; *P* = 0.51)	Did not observe any patient deaths; however, patients with lung infection and multiple organ dysfunction syndrome were excludedNo pronounced AEs were observed within 6 months of follow-up after the treatment
Zhang, 2022^50^	Retrospective	2014–2019	*n* = 196, corticosteroid monotherapy*n* = 25, etanercept + corticosteroids*n* = 21, IVIG + corticosteroids	etanercept + corticosteroids had lower actual mortality than corticosteroid monotherapy and IVIG + corticosteroids, respectively (0% vs. 6.63% and 4.76%)SMR for the etanercept + corticosteroids, IVIG + corticosteroids and corticosteroid monotherapy treatment groups were 0 (95% CI 1.80–3.59), 0.30 (95% CI 0.68–6.22) and 0.71 (95% CI 0.83–2.64), respectively. The etanercept + corticosteroids group showed a significantly lower SMR (*P* = 0.006)	Etanercept + corticosteroids showed a lower incidence of AE with GI haemorrhage vs. corticosteroid monotherapy, especially in patients with TEN (*P* = 0.001)Etanercept + corticosteroids group had higher average SCORTEN scores and total BSA involvement; however, no mortality was noticed in this group
				Etanercept + corticosteroids showed reduced skin healing time [12.0 (8.5–14.0), median days (IQR)] vs. scorticosteroid monotherapy [13.0 (10.0–18.0)] and IVIG + corticosteroids [13.5 (10.0–19.5)]; *P* = 0.004 and *P* = 0.01, respectively)	
Cao, 2023^51^	Meta-analysis	1990–January 2023	*n* = 31, infliximab*n* = 90, etanercept*n* = 4, adalimumab	Infliximab; *n* = 3 SJS/TEN overlap, *n* = 28 patients TEN. Actual mortality; 33.3% and 17%Etanercept; *n* = 17 patients SJS, *n* = 9 SJS/TEN overlap, *n* = 64 TEN MR 0%, 0% and 12.5%Adalimumab; *n* = 4 TEN; MR 25%Etanercept significantly reduced hospitalization time compared with nonetanercept treatments (WMD −5.30; 95% CI −8.65, −1.96; *P* = 0.002; *I*^2^ = 0%)Trend toward lower mortality with etanercept, not statistically significant (OR 0.55; 95% CI 0.23–1.33; *P* = 0.19; *I*^2^ = 0%)	More sequelae reported in patients with TEN receiving infliximab than in those treated with etanercept (39.3% vs. 6.4%, *P* = 0.02)

AE, adverse event; BSA, body surface area; CI, confidence interval; CTL, cytotoxic T lymphocyte; GI, gastrointestinal; IQR, interquartile range; IVIG, intravenous immunoglobulin; MR, mortality rate; NMA, network meta-analysis; OR, odds ratio; RCT, randomized controlled trial; RR, relative risk; SAE, severe adverse event; SCAR, severe cutaneous adverse reactions; SCORTEN, Severity-of-Illness Score for Toxic Epidermal Necrolysis; SJS/TEN, Stevens–Johnson syndrome/toxic epidermal necrolysis; SMR, standardized mortality rate; SUCRA, surface under the cumulative ranking curve; TNF-α, tumour necrosis factor alpha; TPE, total plasma exchange; Treg, regulatory T cell; WMD, weighted mean difference. ^a^Articles comparing etanercept alone for SJS/TEN (i.e. when not combined with any other agent) over alternative therapies. ^b^Data as presented in the original study, mean ± SD (range). ^c^Sex distribution not available for one included study.

## Limitations

Of all the evidence discussed, we acknowledge there have been only two controlled prospective trials (one nonrandomized^4^ and one randomized^6^). Many studies, being case reports and case series, lack the statistical power to draw robust conclusions. Furthermore, amalgamated case reports are potentially subject to reporting bias. Some of the studies reported allude to the response of a mixed group of patients with SJS/TEN overlap, as well as TEN. There was also heterogeneity across treatment protocols (e.g. variations in dosing, timing of administration and combination therapies), making it challenging to generalize conclusions.

## Future research recommendations

Further studies are needed to strengthen the evidence base for etanercept in TEN. Ideally, these would be ­double-blinded randomized controlled trials with standardized treatment protocols and outcome measures, allowing for direct comparability of interventions with adequate power and without bias. It would also be important to measure time to mucosal and cutaneous re-­epithelialization as two separate endpoints, which may impact morbidity/­mortality differently (e.g. intubation time). Ultimately, the rarity of the disease considerably hinders the practicality of such a study, which would require agreement in treatment and collaboration on a national or perhaps international level.

## Conclusion

In conclusion, etanercept appears to be an effective treatment for TEN with improved skin re-epithelialization time, reduced duration of hospital stay, and a reduction in the side-effects seen with other systemic treatments such as corticosteroids. Although it would be premature to anticipate the stance that the next UK guidelines might take on etanercept in the treatment for TEN, it is noteworthy that in the US literature, the *Journal of the American Academy of Dermatology* recently referred to etanercept as a ‘game changer’ in the treatment of TEN.^53^ In the interim period, on the basis of the evidence presented herein, we argue that clinicians should be encouraged to use this widely available, relatively cheap and safe medication to optimize the treatment of patients with this devastating and challenging condition.

## Data Availability

The data underlying this article are available in the article.
